# Low frequency repetitive transcranial magnetic stimulation promotes plasticity of the visual cortex in adult amblyopic rats

**DOI:** 10.3389/fnins.2023.1109735

**Published:** 2023-01-19

**Authors:** Jing Zheng, Wenqiu Zhang, Longqian Liu, Maurice Keng Hung Yap

**Affiliations:** ^1^Department of Ophthalmology, West China Hospital, Sichuan University, Chengdu, China; ^2^Department of Optometry and Visual Science, West China Hospital, Sichuan University, Chengdu, China; ^3^School of Optometry, The Hong Kong Polytechnic University, Hong Kong SAR, China

**Keywords:** amblyopia, visual plasticity, repetitive transcranial magnetic stimulation, GABA, perineuronal nets

## Abstract

The decline of visual plasticity restricts the recovery of visual functions in adult amblyopia. Repetitive transcranial magnetic stimulation (rTMS) has been shown to be effective in treating adult amblyopia. However, the underlying mechanisms of rTMS on visual cortex plasticity remain unclear. In this study, we found that low-frequency rTMS reinstated the amplitude of visual evoked potentials, but did not influence the impaired depth perception of amblyopic rats. Furthermore, the expression of synaptic plasticity genes and the number of dendritic spines were significantly higher in amblyopic rats which received rTMS when compared with amblyopic rats which received sham stimulation, with reduced level of inhibition and perineuronal nets in visual cortex, as observed *via* molecular and histological investigations. The results provide further evidence that rTMS enhances functional recovery and visual plasticity in an adult amblyopic animal model.

## Introduction

The visual neural pathways are not fully developed in the early postnatal period of mammals. Besides genetic influences, the neural pathways may be adjusted and altered by visual experience. This experience-dependent visual cortex plasticity decreases rapidly in early life. Little plasticity is observed in adulthood (Hensch, 2005). Monocular and binocular abnormal visual experiences, such as form deprivation and interocular differences in retinal inputs, can cause underdevelopment of vision in the eye receiving the poorer quality input. This condition, known as amblyopia, is clinically defined as a disorder in which the eye shows decreased vision because of abnormal development of the visual pathway in childhood (Birch, 2013), without obvious organic ocular disease. Amblyopia is the most common reason for vision impairment in a single eye among children and adolescents, affecting 1–2% of the total population (Hashemi et al., 2018; Li et al., 2019). The time window when the visual cortex exhibits the greatest plasticity is termed the critical period (Hensch and Quinlan, 2018), after which the plasticity of the visual cortex is receded, making the treatment of adult amblyopia a clinical challenge (Simons, 2005). More recently, it has been shown that visual cortical plasticity in adulthood may be revived with certain pharmacological (Maya Vetencourt et al., 2008; Silingardi et al., 2010) or environmental interventions (Greifzu et al., 2014; Erchova et al., 2017).

Emerging evidence suggests that the inhibitory neural pathways utilizing gamma-aminobutyric acid (GABA) as a neurotransmitter play a key role in the regulation of visual cortical plasticity. The level of GABAergic inhibition in the visual cortex is relatively low at birth, and the critical period begins when inhibition development reaches a certain threshold. The inhibition level is increased due to continual maturation of the pathways, following which a second threshold is reached that triggers the closure of the critical period (Spolidoro et al., 2009; Heimel et al., 2011). The inhibitory interneurons expressing the calcium-binding protein parvalbumin (PV) are the primary targets of visual input from the lateral geniculate body. As the cortex develops, perineuronal nets (PNNs) composed of extracellular matrix (ECM) are continuously deposited around the bodies and the dendrites of PV interneurons and other neurons, restricting the changes in synaptic connections and leading to the structural closure of critical periods. The artificial degradation of chondroitin sulfate proteoglycans (CSPGs), the primary component of ECM, may reactivate cortical plasticity (Pizzorusso et al., 2002; Heimel et al., 2011).

Invented in 1985, transcranial magnetic stimulation (TMS) is one of several non-invasive brain stimulation (NIBS) modalities (Polanía et al., 2018). How a magnetic pulse influences neuronal activity is described elsewhere (Hallett, 2007). Repetitive TMS (rTMS) refers to the continuous stimulation on the local cortex with a fixed frequency and intensity, which induces persistent current in the brain and promotes synaptic long-term potentiation (LTP) or long-term depression (LTD) (Hallett, 2007).

Previous studies have shown that applying different rTMS modes to particular brain regions in animals may alter neuronal activity by up or downregulating the inhibition level in the cortex, promoting the recovery of some brain-related disorders (Hoppenrath et al., 2016; Jazmati et al., 2018; Tan et al., 2018; Wu et al., 2018). The clinical application of rTMS in neuropsychiatric diseases, such as severe depression, rehabilitation exercise after stroke, schizophrenia, and drug addiction, is becoming more common. In the context of vision, it has been shown that both high- and low-frequency rTMS applied to the visual cortex temporarily improved contrast sensitivity in the adult amblyopic eye (Thompson et al., 2008). Multiple applications of continuous theta-burst stimulation (cTBS), a specific type of rTMS with high frequency and short stimulation time, over 5 days, appear to improve high spatial frequency contrast sensitivity in the adult amblyopic eye. These improvements were stable, lasting over 78 days, suggesting that rTMS may produce long-lasting effects on the amblyopic visual cortex (Clavagnier et al., 2013). Another study showed a temporary and significant improvement in visual and stereoscopic acuities in patients with amblyopia after a single application of cTBS, with decreased interocular suppression (Tuna et al., 2020). Other forms of NIBS involving low currents, such as anodal transcranial direct current stimulation (a-tDCS), have also been shown to improve contrast sensitivity in the adult amblyopic eye (Spiegel et al., 2013).

Although these studies suggest that rTMS may promote improvements in certain visual functions in the adult amblyopic eye, little is known about the mechanisms involved. We speculate that in adult amblyopia, rTMS alters the activity of the visual cortex *via* regulation of genes and inhibitory neural pathways involved in plasticity, to cause a change in visual function. We conducted a series of experiments using an adult rat amblyopia model to further our understanding of changes in the molecular and structural mechanisms associated with rTMS application.

## Materials and methods

### Animals

**FIGURE 1:**
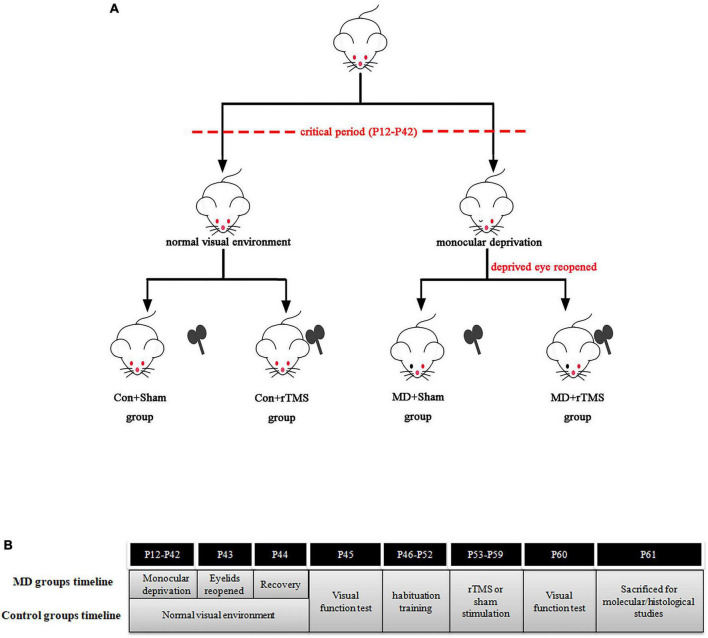
**(A)** The four groups in this study. **(B)** Timelines of monocular deprivation (MD) groups and control groups.

A total of 63 Wistar rats of both genders were used in this study which was approved by the Animal Ethics Committee of Sichuan University and adhered to the guidelines for the Use of Animals in Ophthalmic and Vision Research. The rats were kept in the Animal Experiment Center of West China Hospital under a 12/12 h light/dark cycle and 20–23^°^Cambient temperature, with *ad libitum* access to water and food. The pups lived with their mothers until weaning at P21. The animals were divided into four groups using a table of random numbers: control animals treated with sham stimulation (Con+Sham, *n* = 16), monocularly deprived animals treated with sham stimulation (MD+Sham, *n* = 16), control animals treated with rTMS (Con+rTMS, *n* = 15), and monocularly deprived animals treated with rTMS (MD+rTMS, *n* = 16). The experimental timelines of four groups are shown in Figure 1.

### Monocular deprivation

The animals were anesthetized with 1.25% avertin (2,2,2 tribromoethanol; 10 mL/kg) by intraperitoneal injection at P12. Right upper and lower eyelid margins of 1 mm each were excised, and the eyelids were subsequently stitched shut with 6-0 silk thread. Chlortetracycline was applied on the eyelid closure every day until complete cicatrization. The eyelids were reopened at P43 followed by 24 h recovery to undergo visual function testing before the rTMS treatment. Rats showing lid reopening or infection of the eyelid during MD were excluded from the experiments and not included in the statistical analysis. Control animals also underwent anesthesia and eyelids suture with immediate opening at P12 and P43.

### rTMS

A figure-of-eight coil with a diameter of 40 mm was used to deliver rTMS to conscious rats through an RT-100 Stimulator (JJWF-medicine, Chengdu, China). Following a previously described technique (Castillo-Padilla and Funke, 2016), the rat’s body was wrapped with a soft towel, and its sides were held lightly by placing the index finger on its cheek gently to fix the head. The low-frequency 1 Hz mode with a total of 600 pulses for 10 min was selected, and the stimulation intensity was applied with 25% of maximal machine output below the motor threshold. The motor threshold (MT) was measured as described elsewhere (Fujiki et al., 2020). The stimulator intensity was reduced on motor cortex of rats gradually by 1% of maximum stimulator output (MSO) until half of the motor-evoked potentials under certain intensity higher than 50 μV were produced, and this intensity was recorded as the MT. The average MT of five conscious rats was 28 ± 2.24% MSO. During active stimulation, the center of the coil was placed 5 mm above the skin of the rat’s occiput. For sham stimulation, the distance between the coil and the rat’s head was increased to 15 cm. The stimulation regime was conducted every day on each animal between 6:00 p.m. and 8:00 p.m. for 7 days. To aid adaptation to the environment where rTMS was applied, the animals experienced handling habituation training with the stimulation side of the coil facing the opposite direction at 6:00 p.m. for 7 days before sham or active stimulation. After acclimatization, the stressful defecation/urination in animals was noticeably reduced.

### Visual evoked potentials (VEPs)

The animals in each group underwent flash VEP (fVEP) testing before and after treatment (at P45 and P60) to assess individual objective visual functions. After 30 min of dark adaptation, the animals were anesthetized with 1.25% avertin (10 mL/kg) by intraperitoneal injection. A visual electrophysiological apparatus (IRC Medical Equipment, Chongqing, China) was used following the International Society for Clinical Electrophysiology of Vision (ISCEV) standard. Electrodes were placed according to the Freeman and Sohmer (1995) and Yi et al. (2020) methods. The silver needle recording electrode was placed under the skin at the midpoint between the ears, the reference electrode was placed in the oral cavity, and the ground electrode was implanted into the tail. During flash stimulation, the unrecorded eye was occluded with a black cloth. The parameters of the flashing light were as follows: frequency of 1 Hz, flash intensity of 3.0 cd/m^2^ and band-pass filtered from 1 to 100 Hz with the superposition enforced 100 times. The amplitude of P1 (from negative N1 wave through positive P1 wave peak) was recorded, and then the contralateral vs. ipsilateral value (C/I value, contralateral value: the amplitude of P1 derived from the deprived eyes in MD rats or the right eyes in control rats; ipsilateral value: the amplitude of P1 derived from the non-deprived eyes in MD rats or the left eyes in control rats) was calculated and analyzed. The C/I value reflects ocular dominance (OD) of the preferences of the neurons in the visual cortex (Sawtell et al., 2003).

### Visual cliff test

A modification of the previously published cliff apparatus (Mazziotti et al., 2017; Castaño-Castaño et al., 2019) was applied to evaluate the depth perception of rats before and after treatment (at P45 and P60). The cliff apparatus consisted of an upper and a lower part made of polymethyl methacrylate with the open side directed upward. The four inside walls of the upper box (84 × 53 × 41 cm) were covered with black matte cardboard, and the floor was divided into two regions of equal size (42 × 53 cm). Underneath one region, a checkerboard was made up of black and white squares (3 × 3 cm), defined as the “up” zone (Figure 3A). Another transparent region was the “down” zone, and the checkerboard was placed directly under it at 30 cm to create a cliff. Two incandescent lamps were placed below two patterned checkerboards, respectively, on the bottom of the lower box (84 × 53 × 34 cm) to light up the surfaces of both zones with similar dim brightness. The luminous intensity at the height of rat eyes was measured by a luxmeter (PM6612, PeakMeter, China) set at approximately10 lux. Before the experiment, the animals were acclimated to the room for 30 min. Then, the animals were initially placed at the junction of the two zones and allowed to freely explore the surfaces for 5 min. The rats’ movement track was recorded using an infrared camera (8QS3, Xiaomi, China) during the experiment. Between the trials, the zones surface was cleaned with 15% alcohol. The time spent on the respective zones was recorded manually, and then the discrimination index was calculated using the formula: $\frac{\text{t}\,\left(\text{U}\right)-t\,\left(D\right)}{t\,\left(T\right)}$. t(U): time on “up” zone; t(D): time on “down” zone; t(T): total time.

### qRT-PCR

A total of 16 rats (*n* = 4/group) were sacrificed after treatment for synaptic plasticity gene array analysis. Following deep anesthesia with 1.25% avertin (10 mL/kg) by intraperitoneal injection, the rats were decapitated, and the brain was rapidly removed and placed on ice. According to the rat brain atlas (Paxinos and Watson, 2007), the binocular zone in the primary visual cortex (V1B) contralateral to the deprived eye in MD rats or contralateral to the right eye in control rats was dissected carefully. The total RNA of tissues was isolated and purified using TRIzol Plus RNA Purification Kit (Invitrogen). Subsequently, reverse transcription was conducted using the QuantiNova Reverse Transcription Kit (Qiagen). According to the manufacturer’s guidelines, gene expression alterations were assessed using a synaptic plasticity gene array (RT^2^Profiler PCR Array Rat Synaptic Plasticity, Cat. no. 249950, Qiagen) and QuantiNova SYBR Green PCR Kit (Qiagen).

The classification and gene array panels are described in Supplementary Table 1. The PCR process was performed on QuantStudio3 system (Thermo Fisher), and the data from all groups were entered into an online system^1^ for analysis. Fold change (FC, gene expression relative ratio) was calculated using the classical ΔΔCq method. The changes in gene expression levels between the groups were considered significant if: (1) the gene expression was up- or downregulated by 50% (FC > 1.5 or FC < 1/1.5); (2) the *p*-value was <0.05 for comparison between groups.

### Western blot

All the rats (*n* = 4/group) were anesthetized by intraperitoneal injection with 1.25% avertin (10 mL/kg) and then decapitated. The brain was carefully removed, and V1B contralateral to the deprived eye in MD rats or contralateral to the right eye in control rats was dissected. Brain tissues were immersed in RIPA lysis buffer (Beyotime), followed by lysis in an Ultrasonic Pulverizer (SCIENTZ, Ningbo, China). The protein in the lysate was quantified by BCA Protein Assay Kit (Beyotime). An equivalent of 20 μg was separated by 10% SDS-PAGE (Epizyme) and transferred to polyvinylidene difluoride (PVDF) membranes (Millipore). Subsequently, the membranes were blocked with 5% skimmed milk in TBS containing 0.1% Tween (TBST, BioFroxx) at room temperature (20–25^°^C) for 2 h, and incubated in primary antibody at 4^°^C overnight: (1) GABA synthase: GAD65, 1:1000 (ab239372, Abcam); GAD67, 1:1000 (ab213508, Abcam); (2) GABA transporters: VGAT, 1:1000 (131002, Synaptic System); (3) GABA receptors: GABAAα1, 1:1000 (ab252430, Abcam); GABAAα3, 1:2000 (12708-1-AP, Proteintech); (4) Neurotrophin: BDNF, 1:1000 (AB108319, Abcam); (5) Synapse-related proteins: Syntaxin, 1:1000 (ab272736, Abcam); PSD95, 1:2000 (ab238135, Abcam); (6) reference protein protein glyceraldehyde-3-phosphate dehydrogenase (GAPDH) (MA5-15738, Invitrogen). The blots were washed the next day with TBST (3 × 10 min), incubated with secondary antibody (anti-rabbit, 1:5000, ab6721, Abcam; anti-mouse, 1:5000, 511203, Zenbio) at room temperature for 1 h, then washed in TBST (3 × 10 min). Finally, the blots were immersed in ECL Chemiluminescent Substrate (Biosharp) and visualized using ChemiDoc MP system (Bio-Rad). The gray scale value of the proteins was analyzed using ImageJ (NIH), and the ratio of the target protein to internal reference protein GAPDH was calculated.

### Immunofluorescence

After anesthesia using intraperitoneal injection with 1.25% avertin (10 mL/kg), rats were transcardially perfused with 150 mL of 0.9% saline solution, followed by 100 mL of 4% paraformaldehyde. The brains were removed and immersed in 4% paraformaldehyde at 4^°^C overnight, and then immersed in 30% sucrose solution until they sank to the bottom. The rats’ occipital lobes were cut coronally into 20 μm-slices using a cryostat (Leica) and mounted on slides. The slices were from 2 to 4 sections of an animal, and 3–4 animals (Con+Sham, *n* = 4; MD+Sham, *n* = 4; Con+rTMS, *n* = 3; MD+rTMS, *n* = 4) were used for each group. After drying, the slides were blocked in phosphate-buffered saline (PBS) containing 0.1% Triton X-100 (Solarbio) and 5% normal goat serum (Solarbio) at room temperature (20–25^°^C) for 1 h and incubated with primary antibodies parvalbumin (1:100, ab181086, Abcam) and lectin from Wisteria floribunda agglutinin (1:200, L1516, Sigma) at 4^°^C overnight. Wisteria floribunda agglutinin (WFA) binds to glycosaminoglycans of chondroitin sulfate proteoglycans (CSPGs), the main component of PNNs (Miyata et al., 2018). The slices were washed with PBS (3 × 5 min), followed by staining with secondary antibody anti-rabbit (Cy3) (1:500, ab6939, Abcam) and streptavidin (1:200, 4800-30-14, R&D) for 1 h, and then washed in PBS (3 × 5 min). Subsequently, the nuclei were stained with DAPI (Solarbio). The images were captured under a confocal microscope (Nikon) at 4 × magnification to locate the V1B of each slice. High magnification images of V1B were patched together under a 20× objective under the scanning mode of 1 μm-step z-stack. All images were analyzed with ImageJ (NIH) for the quantification of the number of PV-positive neurons and WFA-positive cells and their colocalization by manual counting.

### Golgi staining

We used FD Rapid Golgi Stain Kit (PK401, FD NeuroTechnologies) to observe the dendritic spines of V1B in all the groups. Rats (*n* = 4/group) were anesthetized with 1.25% avertin (10 mL/kg) by intraperitoneal injection and then decapitated. According to the manufacturer’s instructions, the brains were sequentially immersed in a mixture of solution A and B for 2 weeks and then transferred to solution C for 72 h. Subsequently, the occipital lobe was cut into 150 μm-thick slices using a cryostat (Leica), which were allowed to dry naturally at room temperature, stained with a mixture of solution D and E, dehydrated using graded ethanol, clarified in xylene, and mounted with neutral balsam. The images of cerebral hemisphere sections were viewed under a bright field microscope (Nikon) with a 4× objective to locate the V1B (Figure 7A). The pyramidal neurons share typical characteristics: pyramid shape, with a thick apical dendrite extending to the meninx, and some basal dendrites from the bottom extending horizontally. The pyramidal neurons in V1B were detected under a 10× objective (Figure 7B). Finally, we used a 100× oil immersion lens to examine the dendritic spines of pyramidal neurons under the EDF plugin (NIS-Elements Viewer). The pyramidal neurons included in the statistics were from 3 to 5 sections of an animal. The secondary order basolateral dendrites >20 μm of layer II/II pyramidal neurons were captured as described elsewhere (Pizzorusso et al., 2006), and the density of spines was quantified by manual counting and analyzed using ImageJ (NIH). The headless and long filopodia were considered as the immature state of dendritic spines and not marked.

### Statistical analysis

Results are presented as mean ± standard error of the mean (SEM). Repeated measures analysis of variance (ANOVA) was performed to analyze the data from fVEP and visual cliff before and after treatment, followed by the Bonferroni test for paired comparisons. Two-tailed unpaired Student’s *t*-test of the replicate 2^–Δ*Cq*^ values were used for each gene for comparison between two groups. Two-way ANOVA was conducted for protein and histological indicators, followed by the Bonferroni test. The Scheirer-Ray-Hare test was performed for non-parametric data (the PV-positive cell densities in layer IV among four groups). *p* < 0.05 indicated a statistically significant difference. All statistical analyses were conducted using SPSS 26.0 (IBM).

## Results

### rTMS promotes the recovery of objective visual function in amblyopic rats

**FIGURE 2:**
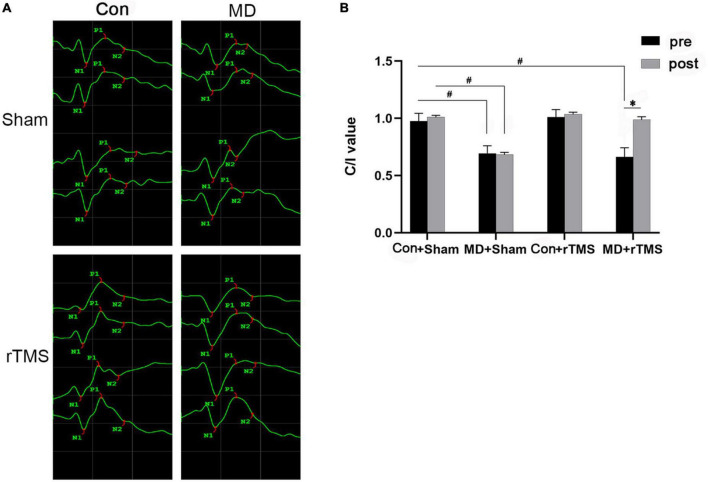
Flash visual evoked potentials (fVEP) before and after treatment in each group. **(A)** The fVEP waveform changes before and after intervention in the four groups. The waveforms from top to bottom in the Con groups were right eye, right eye after intervention, left eye, and left eye after intervention. In monocular deprivation (MD) groups, the waveforms from top to bottom were deprived eye, deprived eye after intervention, non-deprived eye, and non-deprived eye after intervention. **(B)** P1-wave amplitude of fVEP in each group was analyzed before and after intervention, and the data are presented as contralateral vs. ipsilateral value (C/I) value, as assessed by repeated-measures analysis of variance (ANOVA) with Bonferroni’s correction. Following repetitive transcranial magnetic stimulation (rTMS), the C/I value of amblyopic rats improved significantly compared to the previous data. No improvement was observed in amblyopic rats exposed to sham stimulation. Data are expressed as mean ± standard error of the mean (SEM). # denotes significant comparison among MD and Con+Sham group, *p* < 0.001; *indicates significant difference between MD+rTMS pre and after rTMS, *p* < 0.001.

To investigate the possible effect of rTMS on the objective visual function in rats, fVEP was measured *in vivo* before and after intervention in each group (Figure 2A). Among the four groups, the main and interaction effects on the C/I value of the duration of the treatment (time) and the group (treatment) were assessed by repeated-measures ANOVA. The treatment showed a significant main effect [F (3,48) = 80.07, *p* < 0.001], and the main effect of time was also significant [F (1,48) = 67.41, *p* < 0.001] with a significant interaction treatment × time [F (3,48) = 52.51, *p* < 0.001] (Figure 2B). For all the baseline results, the C/I value of MD+Sham and MD+rTMS groups were significantly lower than the Con+Sham group (both *p <* 0.001, repeated-measures ANOVA, Figures 2A, B), indicating that the nerve impulses from deprived eyes responding to flash were diminished in the primary visual cortex compared to the non-deprived eyes in MD rats. Sham stimulation or rTMS did not alter the C/I ratio of control rats compared to the baseline (Con+Sham group: 0.98 ± 0.02 pre vs. 1.01 ± 0.02 after sham stimulation, *n* = 11, *p* > 0.05; Con+rTMS group: 1.01 ± 0.02 pre vs. 1.04 ± 0.02 after rTMS, *n* = 13, *p* > 0.05, repeated-measures ANOVA, Figures 2A, B). The fVEP amplitudes of deprived eyes in the MD group were lower than those of non-deprived eyes before and after 7 days of sham stimulation (C/I value: 0.69 ± 0.02 at baseline, 0.68 ± 0.02 after sham stimulation, *n* = 13, *p* = 0.660). Conversely, after 7 days of rTMS, the amplitudes of deprived eyes in MD rats were similar to those of non-deprived eyes (C/I value: 0.66 ± 0.02 before and 0.99 ± 0.02 after rTMS, *n* = 15, *p* < 0.001). After the intervention, the C/I value of the MD+Sham group differed from the Con+Sham group significantly (*p* < 0.001), while no statistical difference was detected between the MD+rTMS and Con+rTMS groups (*p* = 0.71). These results suggest a promoter role of rTMS in amblyopic rats.

**FIGURE 3:**
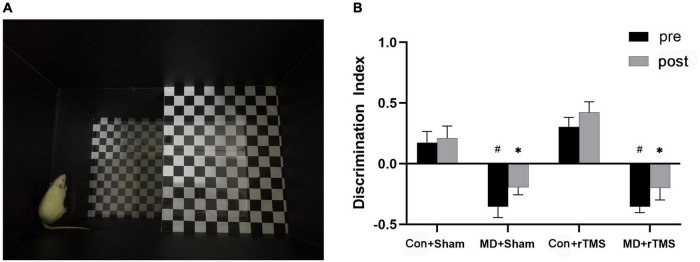
**(A)** An adult rat is exploring the “down” zone in the visual cliff device, while the actual light intensity during behavioral experiments is much dimmer than the environment in this picture. **(B)** Discrimination index was used to quantify the depth perception of rats by repeated-measures analysis of variance (ANOVA) with Bonferroni correction. After repetitive transcranial magnetic stimulation (rTMS), the discrimination index of monocular deprivation (MD) rats did not differ significantly from the baseline and was significantly lower than in Control rats. Data are expressed as mean ± standard error of the mean (SEM). # represents significant comparison among MD and Con+Sham group before receiving stimulation, *p* < 0.05; *indicates significant comparison among MD and Con+Sham groups after receiving the stimulation, *p* < 0.05.

### rTMS fails to improve the depth perception of amblyopic rats

We evaluated the depth perception of rats *via* a visual cliff device (Figure 3A), and the discrimination index of each group was calculated. The main effect on the discrimination index was statistically significant in both treatment [F (3,25) = 20.66, *p* < 0.001] and time [F (1,25) = 7.97, *p* < 0.01], without a significant interaction [F (3,25) = 0.46, *p* = 0.72] (Figure 3B). Regarding the baseline data, the discrimination index in the Con+Sham group was significantly higher than in the MD+Sham and MD+rTMS groups (both *p* < 0.001), indicating that the depth perception of MD rats was impaired markedly compared to control rats. Conversely, no difference was detected between the Con+Sham and Con+rTMS groups. After receiving stimulation, significant differences were detected between the Con+Sham and the two MD groups (both *p* < 0.05). Paired analysis revealed that the discrimination index in each group did not differ significantly before and after stimulation in comparison to themselves. Hence, we deduced that rTMS may not influence advanced visual functions, such as depth perception in amblyopic rats.

### rTMS increases the expression of synaptic plasticity genes in the visual cortex of amblyopic rats

We used qRT-PCR to evaluate the differential expression level of the synaptic plasticity genes of each group.

**FIGURE 4:**
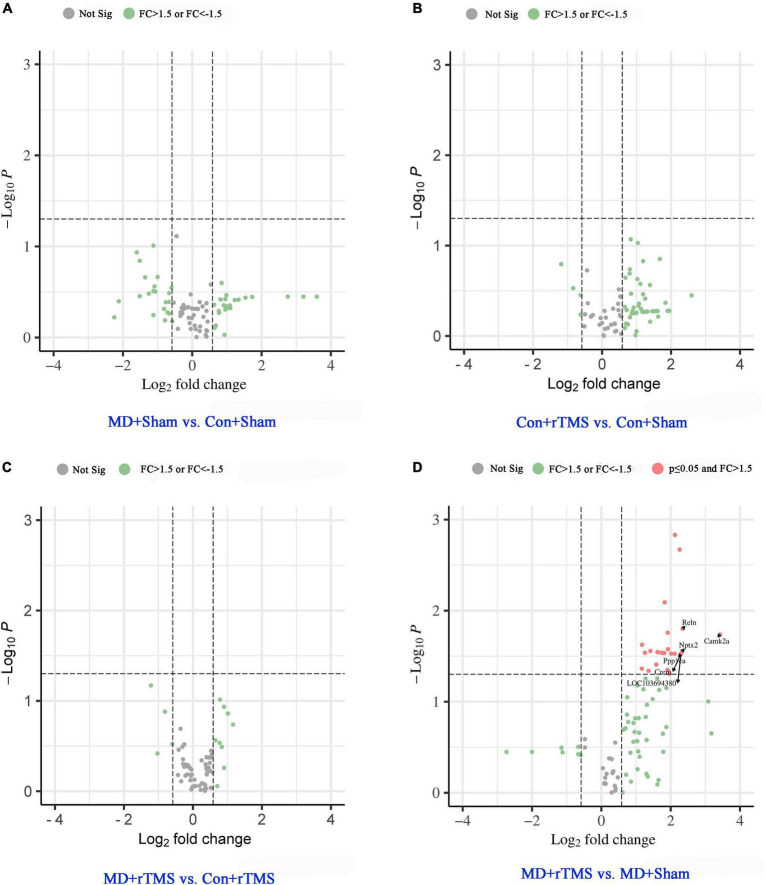
Volcano plots of gene expression changes based on chosen pairwise group comparison. Genes with >50% increased or decreased expression with no significance are shown as green-filled dot, while gray-filled dots indicate gene expression changes between −50 and +50% with no significance. The gene expression changes with *p* < 0.05 (above y = 1.30) were categorized as significant and labeled as red-filled dots. **(A)** Gene expression change between MD+Sham and Con+Sham groups. **(B)** Gene expression change between Con+rTMS and Con+Sham groups. **(C)** Gene expression change between MD+rTMS and Con+rTMS groups. In the above pairwise comparison groups, no statistically significant difference was observed in the gene expression. **(D)** Compared to the MD+Sham group, 25 synaptic plasticity genes were significantly upregulated in MD+rTMS group, and no gene was significantly downregulated. The top six increased synaptic plasticity genes are marked with black arrows. Not Sig, not significant; FC, fold change.

The relative abundance of gene expression is presented in the volcano plot (Figure 4). The abscissa axis shows the fold change in log2 model [FC = 1.5, log2(1.5) = 0.58; FC = 1/1.5, log2(1/1.5) = −0.58]. The *p*-value is converted to a −log10 model in the longitudinal axis [−log10(0.05) = 1.30]. The data points above the horizontal threshold y = 1.30 are statistically significant (*p* < 0.05), while those below the horizontal line are not significant. The green-filled dots in Figure 4 indicate 50% upregulation or downregulation in the genes with no significance, while gray-filled dots indicate gene expression changes between −50 and +50%. Genes with >50% increased expression with significant difference are shown as red-filled dots.

Compared to the Con+Sham group, 22 genes were upregulated and 21 genes were downregulated in the visual cortex in the MD+Sham group, while none of them changed significantly (Figure 4A). After receiving rTMS, 53/84 genes were upregulated and 3/84 genes were downregulated compared to those experiencing sham stimulation in control rats; however, none were altered significantly (Figure 4B). Between groups receiving rTMS, eight genes were upregulated and three were downregulated in amblyopic rats compared to control rats, while none changed significantly (Figure 4C). No genes were significantly downregulated in the MD+rTMS group than in the MD+Shamgroup (Figure 4D), while 25 genes were significantly upregulated (Supplementary Table 2). Among the top six increased synaptic plasticity genes marked with arrows in Figure 4D, the one with the maximal increase in expression was *Camk2a*. Its fold change between the two groups was 10.72 (i.e., normalized expression of this gene in MD+rTMS group was over 10-fold higher than in the MD+Sham group). CAMK2α participates in long-term potentiation (LTP) *via* the regulation of ionotropic glutamate receptors (Küry et al., 2017). The second most upregulated gene was *Reln* (FC = 5.08), which codes for reelin, an ECM molecule playing a major role in layer formation and neuronal migration in the cortex (Tissir and Goffinet, 2003). *Nptx2*, the third most upregulated gene (FC = 5.01), is a member of the immediate early response gene (IEGs) family, mainly involved in glutamate signaling and synaptogenesis (Cho et al., 2008). *Crem* (CAMP-responsive element modulator) and *Ppp1ca* shared the fourth spot with *LOC103694380 (Tnf)*; the FC was 4.92 among the three genes. *Crem* encodes a bZIP transcription factor that binds to the cAMP-responsive element, which might be involved in the regulation of neural migration (Díaz-Ruiz et al., 2008). *Ppp1ca* encodes the protein which is one of the three catalytic subunits of protein phosphatase 1 (PP1) involved in LTP, long-term depression (LTD), and bidirectional plasticity in the mammalian cerebral cortex (Jouvenceau et al., 2006). *LOC103694380 (Tnf)* encodes for tumor necrosis factor-like protein which is involved in the delayed homeostatic increase in the potentiation of open-eye during monocular deprivation (Kaneko et al., 2008), and also contributes to neurogenesis and brain development (Rockstrom et al., 2018).

### rTMS reduces the level of inhibition in V1B of amblyopic rats by decreasing the synthesis of GABA

The proteins were extracted from the contralateral V1B of the deprived eye in amblyopic rats or of the right eye in control rats after treatment. The abundance of the inhibitory neural pathway proteins and synapse-related proteins was analyzed using Western blot. The difference between groups was analyzed using two-way ANOVA.

**FIGURE 5:**
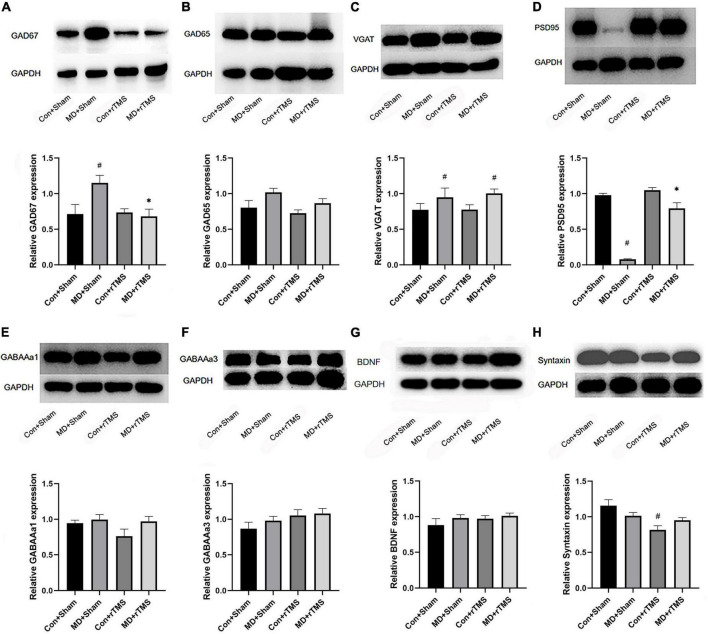
Western blot to detect the expression of the inhibitory neural pathway proteins and synapse-related protein in the binocular area of the visual cortex of rats in each group. **(A–H)** Shows the relative expression of GAD67, GAD65, VGAT, PSD95, GABAAα1, GABAAα3, BDNF, and Syntaxin, respectively. The upper panel of each figure is the electrophoresis band, and the lower is the statistical analysis diagram of the relative ratio of the target protein and the internal reference protein glyceraldehyde-3-phosphate dehydrogenase (GAPDH) in each group, as assessed by two-way analysis of variance (ANOVA). # Indicated a statistically significant difference compared to the Con+Sham group after accepting stimulation, *p* < 0.05;*indicates the significant difference compared to the MD+Sham group following receiving stimulation, *p* < 0.05.

After receiving rTMS, GAD67 levels in the visual cortex of MD rats were significantly decreased compared to those receiving sham stimulation. GAD67 levels in the MD+Sham group were higher than those in the Con+Sham group (Figure 5A). No significant differences were detected in GAD65 levels in the visual cortex between receiving TMS and sham stimulation in MD rats (Figure 5B). Moreover, the VGAT levels in both MD groups were higher than those in the Con+Sham group (Figure 5C), indicating that the transport capacity of GABA is stronger in amblyopic rats than in control rats. rTMS significantly increased the level of PSD95 in amblyopic rats compared to sham stimulation (Figure 5D), indicating that a specific amount of silent state synapses have transformed into a stable state after rTMS (Xu et al., 2020). Furthermore, we determined if rTMS alters the expression of inhibitory receptors; the results showed that neither GABAAα1 nor GABAAα3 was statistically significant among the four groups (Figures 5E, F). Moreover, no significant difference was detected in the BDNF expression among the four groups after stimulation (Figure 5G). Syntaxin, a presynaptic membrane protein widely existing in the central nervous system neurons, was significantly reduced in expression following rTMS compared to sham stimulation among control rats, while no difference was observed between the two MD groups (Figure 5H). This result was consistent with previous studies that low-frequency stimuli may attenuate synaptic transmission efficiency in the control cortex (Fitzgerald et al., 2006). These findings emphasized that 1 Hz rTMS may reactivate the previously inhibited visual cortex in amblyopic rats by reducing the synthesis of GABA in interneurons with the maturity of stable state excitatory synapses.

### rTMS promotes a juvenile-like visual cortex state by reducing the density of PNN-surrounding PV interneurons

**FIGURE 6:**
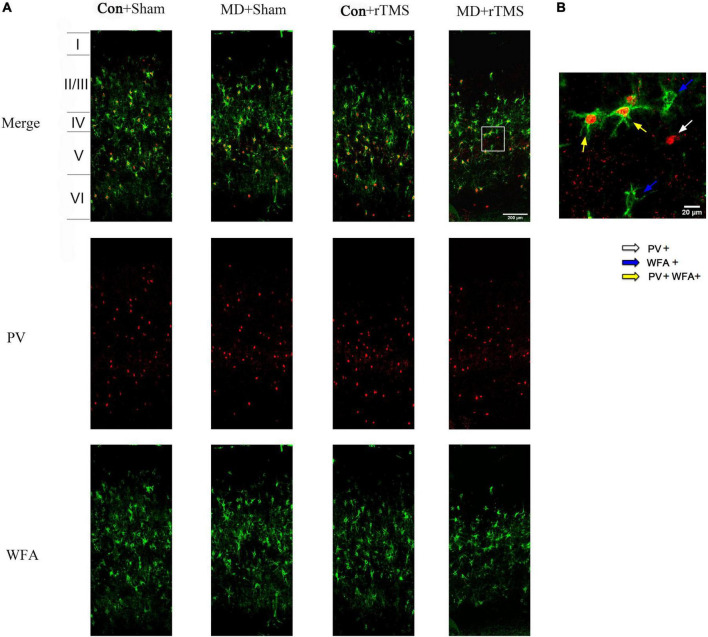
Expression of parvalbumin (PV) and wisteria floribunda agglutinin (WFA)-positive cells in the primary visual cortex (V1B) of the rats in each group. **(A)** Coronal section of V1B in each group. Laminar distribution of PV- and WFA-positive cells are stained with red and green fluorescence, respectively; scale bar: 200 μm. **(B)** A high-magnification view of the area selected by the white frame in panel **(A)**. The white arrow points to PV-positive cells; the blue arrows point to WFA-positive cells; the yellow arrows point to PV/WFA double-labeled cells; scale bar: 20 μm.

To further determine whether rTMS promoted plasticity in the visual cortex of amblyopic rats, we assessed the density of PV interneurons and developed PNNs (Ye and Miao, 2013) by immunofluorescence (Figure 6). We found no difference in the density of PV-positive cells among the four groups with respect to the whole layer or the individual layers of the visual cortex (Supplementary Figures 1A–E). The density of WFA staining cells decreased significantly in the whole visual cortex layer in amblyopic rats receiving rTMS compared to sham stimulation and was also significantly different compared to control rats undergoing rTMS (Supplementary Figure 2A), while no difference was found in layer II/III (Supplementary Figure 2B). In layer IV, the density of WFA-positive cells decreased significantly in the MD+Sham group compared to in the Con+Sham group (Supplementary Figure 2C). And the tendency of WFA density in layers V and VI is similar to that in the whole layer (Supplementary Figures 2D, E). We also analyzed the proportion of PV/WFA double-labeled neurons in PV neurons or WFA-positive cells. In the whole layer, the ratio of WFA/PV double-positive cells to PV-positive cells reduced significantly in amblyopic rats receiving rTMS treatment compared to sham stimulation and in the Con+rTMS group (Supplementary Figure 3A), while no difference was found in layers II/III, IV or V (Supplementary Figures 3B–D). The change of the above ratio in layer VI is consistent with that in the whole layer (Supplementary Figure 3E). We also found no difference in the percentage of WFA-positive cells double-labeled for WFA and PV in the whole layer, layer II/III or layer IV among four groups (Supplementary Figures 4A–C). In layers V and VI, the percentage mentioned above increased in amblyopic rats after rTMS compared to sham stimulation (Supplementary Figures 4D, E). Moreover, this proportion showed a significant increase in the MD+rTMS group than in the Con+rTMS group in layer VI (Supplementary Figure 4E). These results suggest that rTMS promoted juvenile-like plasticity in the visual cortex, which could be attributed to reducing the density of PNNs enwrapping the PV interneurons, a typical characteristic of the immature cortex state (Liu et al., 2013).

**FIGURE 7:**
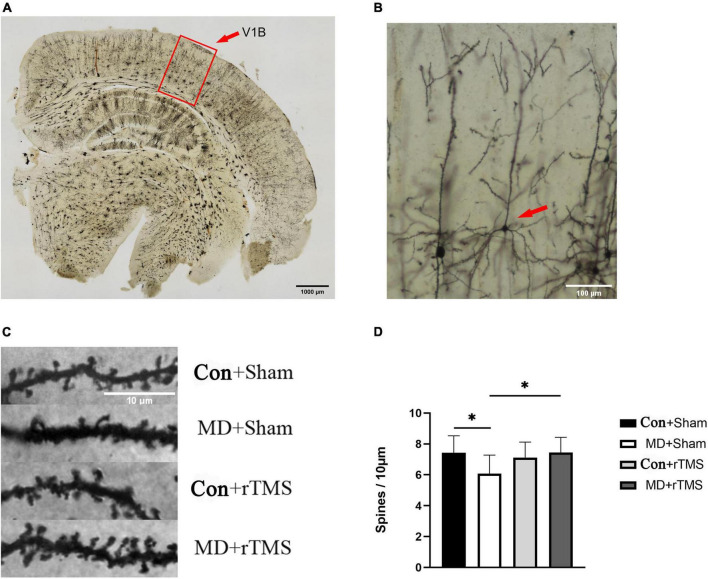
Repetitive transcranial magnetic stimulation (rTMS) effectuates a significant recovery of dendritic spine density in adult amblyopic rats. **(A)** A representative coronal section of the occipital cortex under a low magnification microscope processed *via* Golgi staining. The outline (red) delineates the primary visual cortex (V1B) where dendrites were sampled; scale bar: 1,000 μm. **(B)** A typical morphological pyramidal neuron in the visual cortex is pointed by the red arrow, scale bar: 100 μm. **(C)** Representative dendritic segments from V1B of all four groups. A marked decrease reduction in the dendritic spine density in adult monocular deprivation (MD) rats receiving sham stimulation and an increase in spine density after rTMS; scale bar: 10 μm. **(D)** Quantitative data from all the four groups were assessed by two-way analysis of variance (ANOVA). The dendritic spine density in the MD+Sham group (6.07 ± 1.21) was statistically decreased than that in the Con+Sham group (7.44 ± 1.10). The density in the MD+rTMS group (7.44 ± 0.98) was significantly increased than that in the MD+Sham group (6.07 ± 1.21). *Indicates the significant difference between the two groups, *p* < 0.05.

### rTMS promotes the recovery of dendritic spine density in the primary visual cortex of amblyopic rats

To evaluate whether the effects of rTMS could be mediated by the structural recovery of plasticity of dendritic spines, we assessed the density of secondary order basolateral dendrites on layers II/II pyramidal neurons in V1B contralateral to the deprived eye in MD rats or contralateral to the right eye in control rats by Golgi staining. In rats receiving sham stimulation, the average pyramidal spine density in the visual cortex of MD decreased compared to control rats (Figures 7C, D). After 7 days of rTMS, the dendritic spine density of visual cortical neurons increased significantly in MD rats compared to those undergoing sham stimulation and was close to that of the Con+Sham group (Figures 7C, D). In addition, no significant differences were detected in the spine density between the Con+Sham and Con+rTMS groups, indicating that rTMS did not affect control animals (Figures 7C, D).

Summaries of results indicating statistically significant differences are shown in Tables 1, 2.

**TABLE 1:** Results comparison between control (Con) groups and monocular deprivation (MD) groups.

	Con+Sham	Con+rTMS
MD+Sham	C/I value↓ Discrimination index↓ GAD67↑, VGAT↑, PSD95↓ PNNs density↓ Dendritic spine density↓	C/I value↓ Discrimination index↓
MD+rTMS	Discrimination index↓ VGAT↑	Discrimination index↓

The table shows how the MD groups listed in row title changed when compared with Con groups listed in column title. ↑ Indicates the significant increase / upregulation. ↓ Indicates the significant decrease / downregulation. Con, control; MD, monocular deprivation; Sham, sham stimulation; rTMS, repetitive transcranial magnetic stimulation; C/I value, contralateral vs. ipsilateral value (contralateral value: the amplitude of P1 derived from the deprived eyes in MD rats or the right eyes in control rats; ipsilateral value: the amplitude of P1 derived from the non-deprived eyes in MD rats or the left eyes in control rats); GAD67, glutamate decarboxylase 67 kDa isoform; VGAT, vesicular GABA transporter; PSD95, postsynaptic density 95; PNNs, perineuronal nets.

**TABLE 2:** Results comparison between sham stimulation-treated groups and repetitive transcranial magnetic stimulation (rTMS)-treated groups.

	MD+sham	Con+Sham
MD+rTMS	C/I value↑ 25 synaptic plasticity genes↑ GAD67↓, PSD95↑ PNNs density↓ Dendritic spine density↑	Discrimination index↓ PNNs density↓ Dendritic spine density
Con+rTMS	C/I value↑ Discrimination index↑	Syntaxin↓

The table shows how the rTMS-treated groups listed in row title changed when compared with sham stimulation-treated groups listed in column title. ↑ Indicates the significant increase / upregulation. ↓ Indicates the significant decrease / downregulation. Con, control; MD, monocular deprivation; Sham, sham stimulation; rTMS, repetitive transcranial magnetic stimulation; C/I value, contralateral vs. ipsilateral value (contralateral value: the amplitude of P1 derived from the deprived eyes in MD rats or the right eyes in control rats; ipsilateral value: the amplitude of P1 derived from the non-deprived eyes in MD rats or the left eyes in control rats); GAD67, glutamate decarboxylase 67 kDa isoform; PSD95, postsynaptic density 95; PNNs, perineuronal nets.

## Discussion

This study demonstrated that low-frequency 1 Hz rTMS improved the visual function in adult amblyopic rats, along with molecular and structural changes in the visual cortex. Compared with sham stimulation, rTMS enhanced the restoration of shifted C/I value induced by long-term MD. There was no statistical difference in depth perception between the MD+Sham group and MD+rTMS group.

rTMS-treated MD rats demonstrated increased expression of specific synaptic plasticity genes and decreased levels of the GABA-producing protease in the visual cortex compared to the MD+sham group. This study also demonstrated that rTMS promoted a juvenile-like visual cortex in amblyopic rats by decreasing PNNs and improving the functional synaptic connections made by dendritic spines. These results suggest that rTMS has a beneficial effect on the plasticity of the visual cortex in an adult rat model of amblyopia.

Previous research has demonstrated that rTMS at low frequencies (0.2–1 Hz) induces decreases in brain excitability, while high frequency stimulation (5 Hz) activates brain (Hallett, 2007). However, the specific consequences of different stimulation frequencies on the cortex depend on the cortex’s level of excitability. The adaptive changes of “regulatory threshold” (a specific level of postsynaptic response, above which LTP will be induced, and below which LTD will be induced) to synaptic activity ensure the stability of neural activity in the brain (Bienenstock et al., 1982). In other words, increased postsynaptic activity raises the regulatory threshold and causes LTD. On the contrary, LTP occurs when a sustained decline in postsynaptic activity lowers the regulatory threshold. The theory of homeostatic plasticity revolves around the deliberate modification of synaptic strength to preserve physiological balance. The application of 1 Hz rTMS to the excitatory-preconditioned cortex reduced the excitability below the baseline levels for >20 min. In contrast, cortical excitability increased for at least 20 min after 1 Hz rTMS on the inhibitory-preconditioned cortex (Siebner et al., 2004). The neurons driven by the amblyopic eye in the visual cortex are suppressed (Barnes et al., 2001). Therefore, we used a low-frequency mode of rTMS to change the ratio of excitation to inhibition in amblyopic rats. To rule out the effects of sound, thermal stimulation, and the stressful state brought on by the physical handling of the rats during the trials, we set up sham stimulation groups as controls.

Long-term MD during the critical period results in a weakened response of binocular cells to the deprived eye, followed by an increased response to the non-deprived eye, i.e., the ocular dominance shift (Hofer et al., 2006). Based on fVEP, the C/I values were significantly decreased in MD rats compared to controls in the present study, suggesting a reduced input from deprived eyes to the primary visual cortex or an increase of the non-deprived eye input. After rTMS, the C/I values increased to near normal levels in MD rats, suggesting the restoration of ocular dominance plasticity in the adult primary visual cortex. The rTMS-induced changes in the visual cortex are in line with earlier clinical studies (Thompson et al., 2008; Clavagnier et al., 2013; Tuna et al., 2020).

In addition to binocular integration, certain neurons in the visual cortex of rodents also feature binocular disparity, a function which underlies stereopsis (La Chioma et al., 2020). Stereopsis is processed by the high-order extrastriate cortex (Henriksen et al., 2016). In the treatment of amblyopia, visual acuity improvement after treatment is often not accompanied by a concurrent improvement in contrast sensitivity and stereoacuity (Wallace et al., 2011; Jia et al., 2022). In this study, depth perception was impaired in the MD rats compared to controls, as demonstrated by the visual cliff apparatus. We observed that rTMS did not affect depth perception in our MD rats. However, Tuna et al. (2020) reported that the use of continuous theta burst rTMS could improve stereopsis in adult amblyopia. Zhong et al. (2021) reported that a combination of long-term rTMS with simultaneous sensory training induced sustainable changes in plasticity in the rat’s somatosensory cortex compared with a single session. We speculate that the recovery of binocular visual functions may require a longer-lasting rTMS mode such as cTBS, with supplementary treatments such as perceptual learning for instance.

Among a series of significantly elevated synaptic plasticity genes in our rTMS-treated amblyopic rats, immediate early response genes (IEGs) were predominant. IEGs respond rapidly to various extracellular stimuli, such as growth factors, mitogens, immune, and stress, and more than 100 genes are classified as IEGs (Minatohara et al., 2015). These genes also induce neuronal activity-dependent adaptive changes in downstream pathways, modulate synaptic activity, and may be a gateway controlling synaptic plasticity (de Bartolomeis et al., 2017). Other studies have also shown that rTMS enhanced cortical function by regulating the expression of IEGs in several brain-related disorders, such as Parkinson’s disease (Cacace et al., 2017) and depression (Sun et al., 2015).

In this study, GAD67 was significantly decreased in the visual cortex of amblyopic adult rats receiving rTMS. Previous studies have demonstrated that reducing the synthesis and transport of GABA in the adult visual cortex is crucial for remodeling plasticity (Harauzov et al., 2010; Li et al., 2018). The administration of picrotoxin (PTX, a GABAA receptor antagonist) or 3-mercaptopropionic acid (MPA, a competitive inhibitor of GADs) to the adult rat visual cortex promoted the recovery of ocular dominance plasticity through reducing inhibitory neurotransmission pharmacologically (Harauzov et al., 2010). Cyclin-dependent kinase 5 (Cdk5) forms a complex with GAD67 in the visual cortex, and knockdown of Cdk5 may reduce GAD expression and restore juvenile-like OD plasticity in adult mice (Li et al., 2018). Trippe et al. (2009) reported that 1 Hz rTMS reduced the expression of GAD67 in the visual cortex of adult rats rapidly (within 2 h after the application of rTMS). The change of GAD67 reversed 1 day after rTMS and the increase lasted up to the 7th day. In Trippe et al. (2009) study, the chronic effect on GAD67 in control rats was evaluated after a 1 day single session of 1 Hz rTMS. However, our rats received consecutive stimulation for 7 days. The compromised visual cortical neurons of amblyopic rats may also respond differently to rTMS compared with control rats.

In this study, postsynaptic density 95 (PSD95) significantly decreased in amblyopia compared to controls, which was altered by rTMS. A previous study reported the upregulation of PSD95 by low-frequency rTMS in Alzheimer’s Disease (Pang and Shi, 2021) and spatial cognition impairment (Ma et al., 2014). PSD95, an abundantly expressed scaffold protein, can recruit multiple components in the signaling pathway, such as neurotransmitter receptors and ion channels. Then PSD95 make these components interact or combine to form a complex to regulate signal transduction (Wegner et al., 2018). It is essential for the formation of excitatory synapses (Xu et al., 2020). This is consistent with our findings that the increase of PSD95 was accompanied by a marked increase in excitatory dendritic spine density.

Our study has several limitations. First, we used a 40 mm small coil whose field of stimulation was larger than the visual cortex of rats and might lead to multiple areas affected. It was not comparable to the regional specificity of local stimulation to the human cortex. The second limitation is that the period of observation of the treatment effects was relatively short. As a result, we do not know how persistent the changes associated with the rTMS treatment are. Furthermore, the monocular deprivation method used in this study simulates form-deprived amblyopia only. Other types of amblyopia such as strabismic and ametropic amblyopia which are more common in humans are less often studied in an animal model and could be the focus of future studies.

Our study demonstrated that rTMS promoted the reinvigoration of plasticity of the amblyopic visual cortex in a rat model. It is unknown whether rTMS has other effects on the synaptic environment besides rebalancing the excitatory and inhibitory states. Future studies should examine if other forms of plasticity could be restored by rTMS and clarify the relationship between these mechanisms and the inhibitory neural pathways. Understanding the underlying mechanisms will strengthen the evidence for the use of rTMS in the clinical treatment of adult amblyopia.

## Conclusion

Application of low-frequency rTMS resulted in the recovery of visual function in an adult rat model of amblyopia, presumably arising from a reduction in the level of inhibition in the visual cortex.

## Data availability statement

The original contributions presented in this study are included in this article/Supplementary material, further inquiries can be directed to the corresponding author.

## Ethics statement

The animal study was reviewed and approved by the Animal Ethics Committee of Sichuan University.

## Author contributions

JZ: conceptualization, methodology, investigation, and writing–first draft. WZ: conceptualization, data curation, and project administration. LL: writing–review and editing, data interpretation, and supervision. MH: writing–review and editing and data interpretation. All the authors have read and approved the final manuscript.

## Footnotes

1
https://geneglobe.qiagen.com/us/analyze
