# Supplementary material for: Low frequency repetitive transcranial magnetic stimulation promotes plasticity of the visual cortex in adult amblyopic rats
Source: Front Neurosci. 2023 Jan 19;17:1109735. doi: 10.3389/fnins.2023.1109735 (PMC9892759; doi:10.3389/fnins.2023.1109735)
Supplement: Supplementary file 1 [file Data_Sheet_1.docx]

Supplementary Material

## Supplementary Figures and tables

##
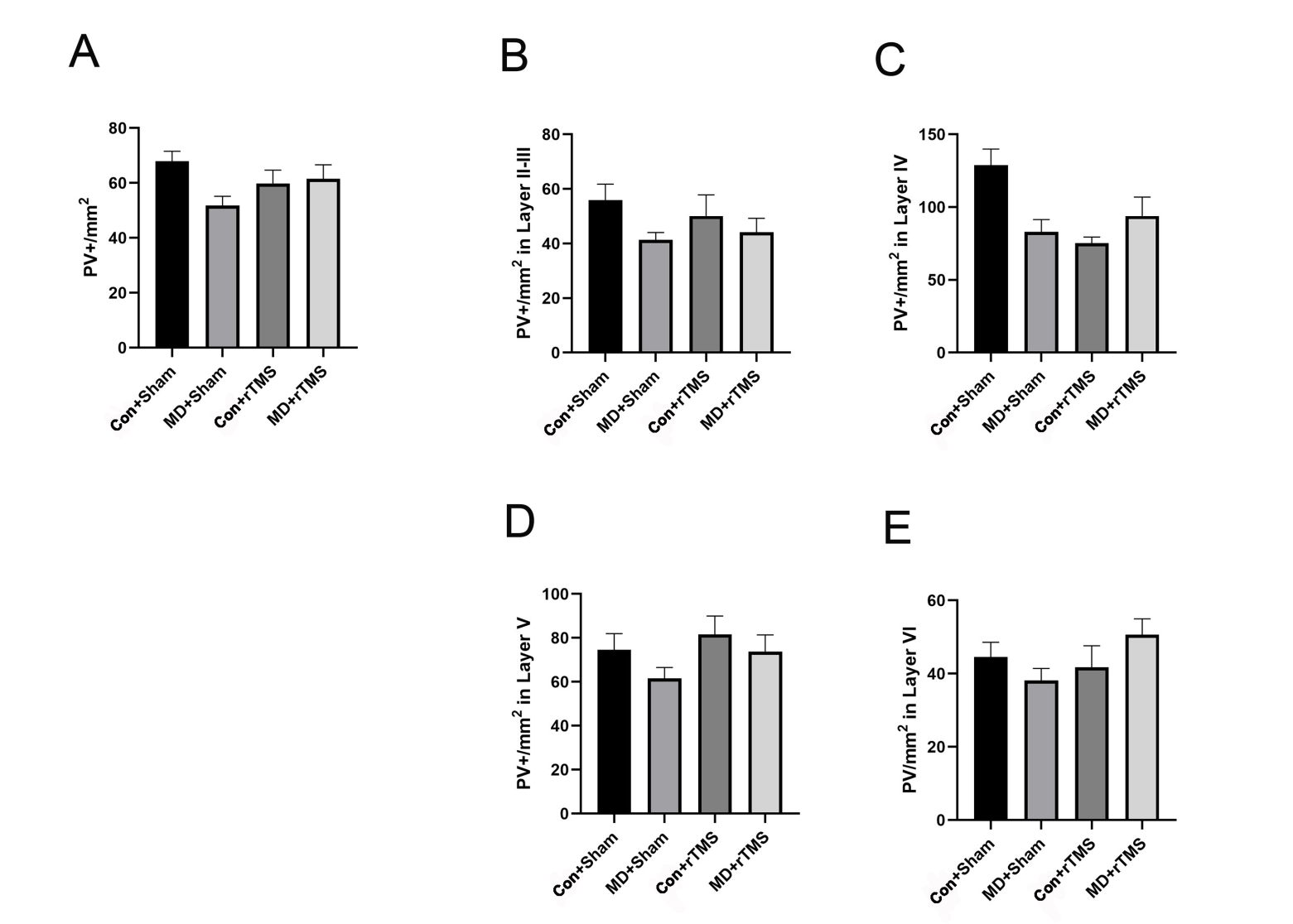
Supplementary Figure 1. Quantitative analysis of the comparison of PV-positive cell densities in the V1B in each group by two-way ANOVA. (A), No significant difference was observed in the density of PV-positive cells in the whole cortex among the four groups, while the density in the MD+rTMS group was increased compared to the MD+Sham group. (B–E), No significant difference was detected in the density of PV-positive cells in layers II/III, IV, V, and VI in each group, respectively. (C), The equal variances were not assumed in layer IV, such that the Scheirer-Ray-Hare test was used.

**
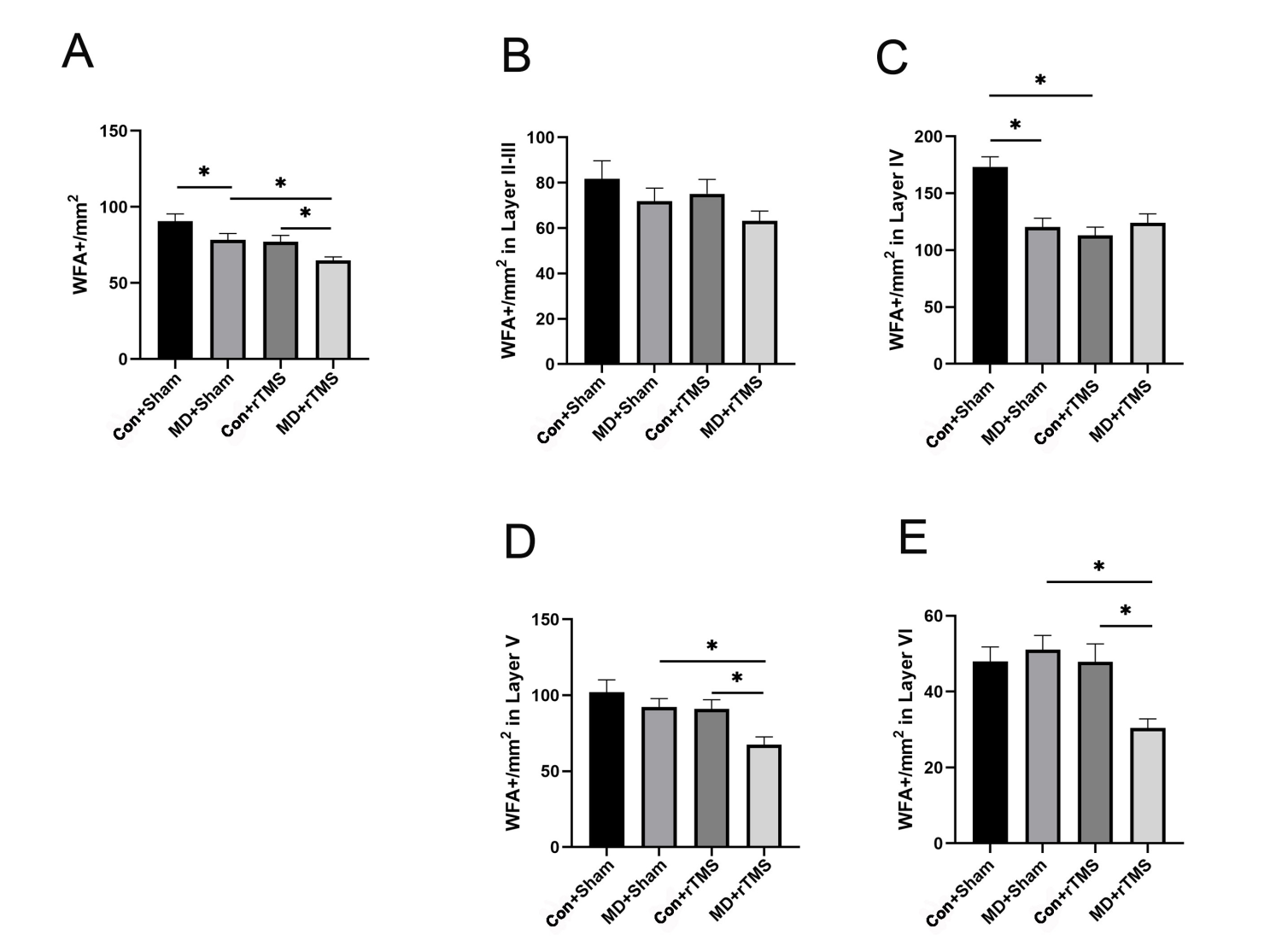
**

## Supplementary Figure 2. Quantitative analysis of the comparison of WFA-positive cell densities in the V1B among each group in the overall layer and layers II/III, IV, V, and VI, respectively, by two-way ANOVA.* indicates a significant difference between the two groups, *p*<0.05.


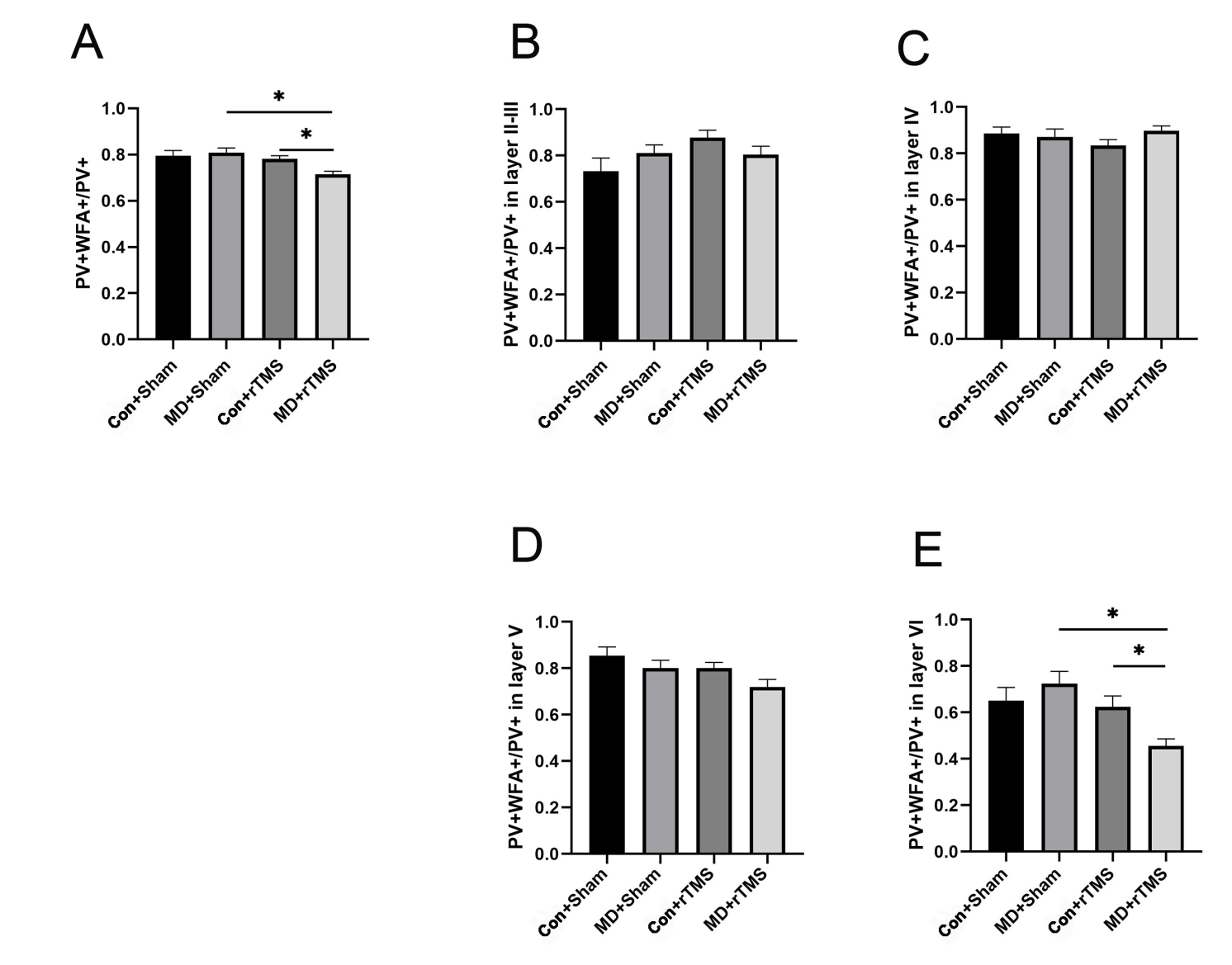


## Supplementary Figure 3. Statistical analysis of the comparison of the proportion of PV/WFA double-labeled neurons in PV-positive cells in the V1B among each group in the overall layer and layers II/III, IV, V, and VI, respectively by two-way ANOVA. * indicates a significant difference between the two groups, *p*<0.05.


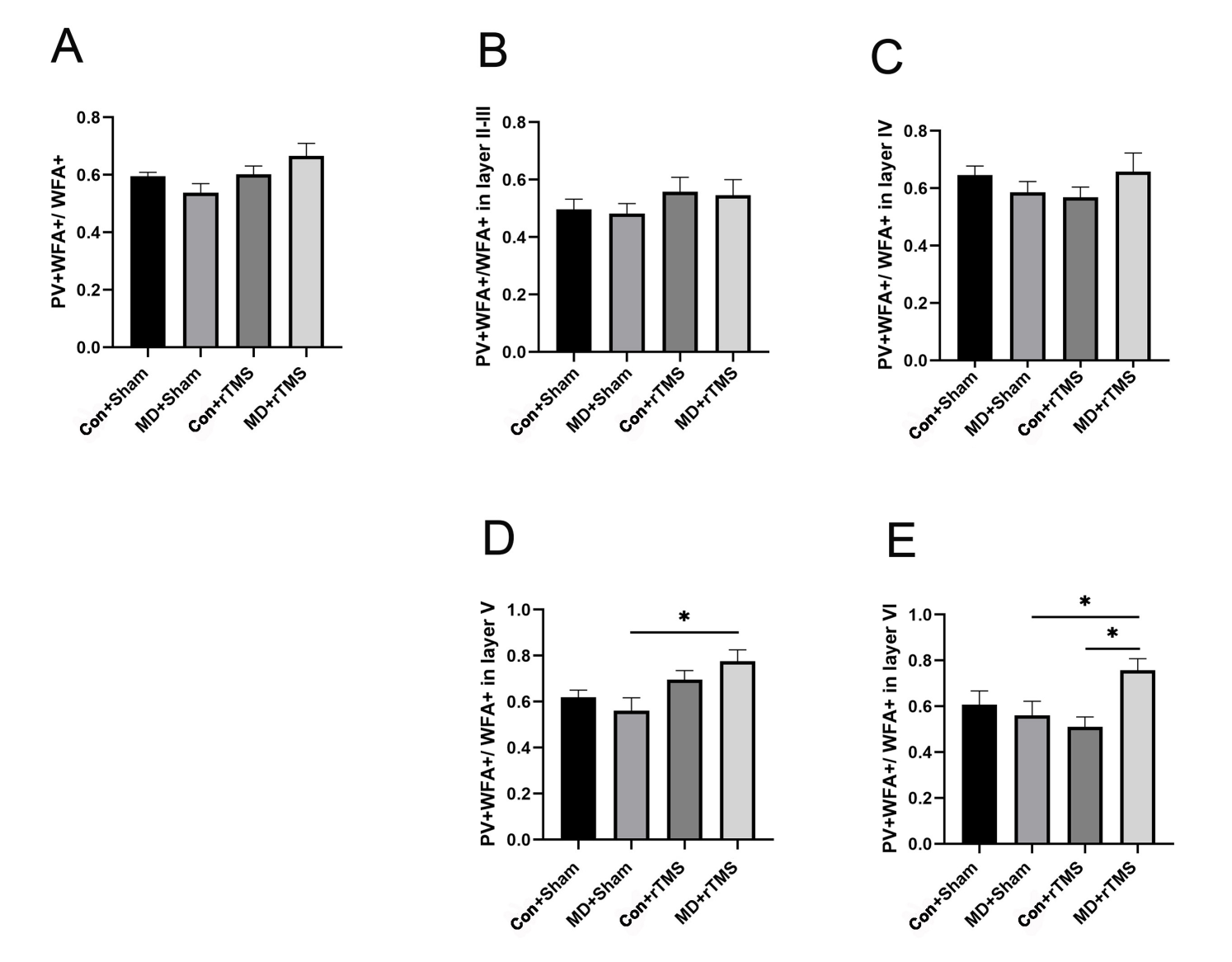


**Supplementary Figure 4.** Statistical analysis of the comparison of the percentage of PV/WFA double-labeled neurons in WFA-positive cells in the V1B among each group in the overall layer and layers II/III, IV, V, and VI, respectively by two-way ANOVA. * indicates a significant difference between the two groups, *p*<0.05.

**Supplemental Table 1.** Classification of synaptic plasticity genes in array panel (some genes were involved in multiple categories)

| **Immediate-Early Response Genes (IEGs)** | **Late Response Genes** | **Long Term Potentiation (LTP)** | **Long Term Depression (LTD)** | **Cell Adhesion Molecules** | **Extracellular Matrix (ECM) Molecules** | **CREB Cofactors** | **Neuronal Receptors** | **Postsynaptic Density (PSD)** | **Other Synaptic Plasticity Genes** |
| --- | --- | --- | --- | --- | --- | --- | --- | --- | --- |
| *Arc,*  *Bdnf,*  *Cebpb,*  *Cebpd,*  *Creb1,*  *Crem,*  *Egr1,*  *Egr2,*  *Egr3,*  *Egr4,*  *Fos,*  *Homer1,*  *Jun,*  *Junb,*  *Klf10,*  *LOC103694380*  *(Tnf)*  *Mmp9,*  *Nfkb1,*  *Nfkbib (Trip9),*  *Ngf,*  *Nptx2,*  *Nr4a1 (Nur77),*  *Ntf3,*  *Pcdh8,*  *Pim1,*  *Plat (tPA),*  *Rela,*  *Rgs2,*  *Rheb,*  *Srf.* | *Inhba,*  *Synpo.* | *Adcy1,*  *Adcy8,*  *Bdnf,*  *Camk2a,*  *Camk2g,*  *Cdh2 (N-cadherin) Cnr1,*  *Gabra5,*  *Gnai1,*  *Gria1,*  *Gria2,*  *Grin1,*  *Grin2a,*  *Grin2b,*  *Grin2c,*  *Grin2d,*  *Mapk1 (Erk2),*  *Mmp9,*  *Ntf4,*  *Ntrk2,*  *Plcg1,*  *Ppp1ca,*  *Ppp1cc,*  *Ppp3ca,*  *Prkca,*  *Prkcg,*  *Rab3a,*  *Ywhaq* | *Gnai1,*  *Gria1,*  *Gria2,*  *Gria3,*  *Gria4,*  *Grip1,*  *Grm1,*  *Grm2,*  *Igf1,*  *LOC103694903*  *(Ppp2ca),*  *Mapk1 (Erk2),*  *Ngfr,*  *Nos1 (bNOS),*  *Pick1,*  *Plat (tPA),*  *Ppp1ca,*  *Ppp1cc,*  *Ppp1r14a (Cpi-17) Ppp3ca,*  *Prkca,*  *Prkg1.* | *Adam10,*  *Cdh2,*  *Grin2a,*  *Grin2b,*  *LOC103694380*  *(Tnf),*  *LOC103694903*  *(Ppp2ca),*  *Ncam1,*  *Pcdh8,*  *Reln.* | *Adam10,*  *Mmp9,*  *Plat (tPA),*  *Reln,*  *Timp1* | *Akt1,*  *Camk2g,*  *Grin1,*  *Grin2a,*  *Grin2b,*  *Grin2c,*  *Grin2d,*  *Mapk1*  *(Erk2),*  *Ppp1ca,*  *Ppp1cc*. | *Ephb2,*  *Gabra5,*  *Gria1,*  *Gria2,*  *Gria3,*  *Gria4,*  *Grin1,*  *Grin2a,*  *Grin2b,*  *Grin2c,*  *Grin2d,*  *Grm1,*  *Grm2,*  *Grm3,*  *Grm4,*  *Grm5,*  *Grm7,*  *Grm8,*  *Ntrk2* | *Adam10,*  *Arc,*  *Dlg4 (Psd95),Gria1,*  *Gria3,*  *Gria4,*  *Grin1,*  *Grin2a,*  *Grin2b,*  *Grin2c,*  *Grm1,*  *Grm3,*  *Homer1,*  *Pick1,*  *Synpo.* | *Kif17,*  *Sirt1* |

| **Supplemental Table 2** 25 genes significantly upregulated in MD+rTMS group than MD + sham group, and the 6 genes with the most increases are marked in red. | | | |
| --- | --- | --- | --- |
| **Gene** | **Fold Change** | ***p*** Value | **Category** |
| *Camk2a* | 10.72 | 0.018306 | Long Term Potentiation (LTP) |
| *Cebpb* | 2.26 | 0.023691 | Immediate-Early Response Genes (IEGs) |
| *Cnr1* | 3.79 | 0.026472 | Long Term Potentiation (LTP) |
| *Crem* | 4.92 | 0.029998 | Immediate-Early Response Genes (IEGs) |
| *Egr3* | 4.79 | 0.002136 | Immediate-Early Response Genes (IEGs) |
| *Gnai1* | 3.00 | 0.039115 | Long Term Potentiation (LTP),  Long Term Depression (LTD) |
| *Grin2d* | 3.35 | 0.029004 | Long Term Potentiation (LTP),  CREB Cofactors, Neuronal Receptors |
| *Grip1* | 2.57 | 0.045809 | Long Term Depression (LTD) |
| *Grm2* | 4.04 | 0.029652 | Long Term Depression (LTD),  Neuronal Receptors |
| *Grm4* | 4.36 | 0.001475 | Neuronal Receptors |
| *Grm7* | 3.51 | 0.029197 | Neuronal Receptors |
| *Grm8* | 3.07 | 0.028552 | Neuronal Receptors |
| *Junb* | 2.39 | 0.029018 | Immediate-Early Response Genes (IEGs) |
| *Ngf* | 4.34 | 0.029807 | Immediate-Early Response Genes (IEGs),  Long Term Depression (LTD) |
| *Nos1* | 3.12 | 0.028655 | Long Term Depression (LTD) |
| *Nptx2* | 5.01 | 0.028081 | Immediate-Early Response Genes (IEGs) |
| *Ntf3* | 3.76 | 0.044851 | Immediate-Early Response Genes (IEGs) |
| *Pim1* | 2.67 | 0.027701 | Immediate-Early Response Genes (IEGs) |
| *Plat* | 3.77 | 0.017474 | Immediate-Early Response Genes (IEGs),  Long Term Depression (LTD),  Extracellular Matrix (ECM) Molecules |
| *Ppp1ca* | 4.92 | 0.029998 | Long Term Potentiation (LTP),  Long Term Depression (LTD),  CREB Cofactors |
| *Reln* | 5.08 | 0.015793 | Cell Adhesion Molecules,  Extracellular Matrix (ECM) Molecules |
| *Kif17* | 3.90 | 0.049462 | Other |
| *Rheb* | 2.25 | 0.043389 | Immediate-Early Response Genes (IEGs) |
| *Srf* | 3.55 | 0.008104 | Immediate-Early Response Genes (IEGs) |
| *LOC103694380 (Tnf)* | 4.92 | 0.029998 | Immediate-Early Response Genes (IEGs),  Cell Adhesion Molecules |
